# A Case of Cannabis-Induced Catatonia and Management With Electroconvulsive Therapy

**DOI:** 10.7759/cureus.43478

**Published:** 2023-08-14

**Authors:** Aditi Sharma, Vinod Sharma

**Affiliations:** 1 Psychiatry, The Wright Center for Graduate Medical Education, Scranton, USA

**Keywords:** cannabis legalization, treatment-resistant catatonia, electroconvulsive therapy (ect), cannabis induced catatonia, cannabis use

## Abstract

This is a case of cannabis-induced catatonia in an 18-year-old Hispanic male with no prior psychiatric history. Shortly after consuming marijuana, the patient experienced catatonic symptoms and demonstrated resistance to several medicinal therapies. Electroconvulsive therapy (ECT) proved to be a useful treatment choice, resulting in significant improvement in symptoms. This example emphasizes the potential dangers of cannabis usage, particularly in susceptible individuals, and underscores the importance of recognizing and treating catatonia as a possible side effect of cannabis use.

## Introduction

Cannabis is one of the world's most widely utilized psychoactive substances [1]. While its recreational and medical properties are well known, growing evidence links it to various psychiatric symptoms such as psychosis, anxiety, and mood disorders. Catatonia caused by cannabis is a rare and under-reported condition. We discuss a case of cannabis-induced catatonia in a young Hispanic boy, emphasizing clinical characteristics, diagnostic problems, and treatment choices. Catatonia is a clinical syndrome defined by a specific set of psychomotor disorders, including immobility, mutism, unwillingness to eat or drink, negativism, and severe psychomotor agitation. There are three subtypes: retarded, excited, and malignant [2]. Mutism, negativism, immobility, echolalia, gazing, and stiffness are symptoms of retarded catatonia [2,3]. Excited catatonia is associated with psychomotor agitation [2,3]. Malignant catatonia is a life-threatening presentation characterized by movement disturbance, autonomic dysfunction, and hyperthermia and can be fatal [3,4]. Catatonia prevalence is reported to be between 7.6% and 38% in the United States, which has been increasing with the increased use of recreational substances [5].

This article was previously presented as a meeting abstract at the American Psychiatric Association 2021 Annual Meeting on May 3, 2021.

## Case presentation

An 18-year-old Hispanic male without any prior psychiatric history was taken to the ED by his mother due to his inability to care for himself and his refusal to eat or drink. His mother reported he had been using marijuana for the last two weeks every day and started exhibiting unusual behaviors like staring at one point, minimal speech, being withdrawn to his room, and sitting in one place for hours without moving, which progressed to being mute, immobile and refusing to eat or drink. On examination, the patient was found to be alert, with a vacant gaze. He did not respond to verbal or tactile stimuli and displayed rigidity in his limbs. He gave initial resistance while repositioning his arms but maintained the posture showing waxy flexibility. He displayed negativism by turning away when asked questions and closing his mouth when offered food or liquids. Bush-Francis Catatonia Rating Scale was used to assess the severity of catatonia. His initial severity score was 25. The rest of the neurological and physical examinations were within normal limits. Extensive laboratory investigations, including a complete blood count, comprehensive metabolic panel, and lipid profile, were within the normal range, as seen in Table 1.

**Table 1:** Laboratory results of the patient. HGB: Hemoglobin; HCT: Hematocrit; MCV: Mean corpuscular volume; PLT: Platelet count; BUN: Blood area nitrogen; eGFR: Estimated glomerular filtration rate; TSH: Thyroid stimulating hormone; T3: Triiodothyronine; T4: Thyroxine; AST: Aspartate aminotransferase; ALT: Alanine transaminase.

Component	Reference Range and Units	Results
WBC	4.00-10.80 K/uL	9.80 K/uL
HGB	14.0-16.8 g/dL	16.1 g/dL
HCT	40.0-48.4 %	46.40%
MCV	82.0-99.5 fL	82.1 fL
PLT	140-400 K/uL	237 K/uL
Lipid profile		
Triglycerides	<200 mg/dL	121 mg/dL
Cholesterol	<170 mg/dL	167 mg/dL
Cholesterol-HDL Ratio	-	3.2
HDL Cholesterol	>34 mg/dL	53 mg/dL
Comprehensive metabolic profile		
Sodium	135-146 mmol/L	140 mmol/L
Potassium	3.5-5.1 mmol/L	4.0 mmol/L
Chloride	98-107 mmol/L	103 mmol/L
CO2	22-32 mmol/L	24 mmol/L
BUN	6-20 mg/dL	17 mg/dL
Creatinine	0.6-1.2 mg/dL	0.9 mg/dL
eGFR	>60	>60.0
Anion Gap	7-15 mmol/L	13 mmol/L
Glucose	70-120 mg/dL	80 mg/dL
Calcium	8.4-10.2 mg/dL	9.5 mg/dL
Magnesium	1.5-2.6 mg/dL	1.8 mg/dL
TSH	0.27-4.2 uIU/mL	1.63 uIU/mL
T4, Free	0.9-1.7 ng/dL	1.16 ng/dL
T3, Free	2.5-4.3 pg/mL	3.2 pg/mL
Liver function tests		
Albumin	3.8-5.0 g/dL	4.7 g/dL
AST (SGOT)	10-50 U/L	22 U/L
ALT (SGPT)	10-50 U/L	23 U/L
Alkaline Phosphatase	0-153 U/L	97 U/L
Bilirubin, Total	0-1.2 mg/dL	0.6 mg/dL

**Figure 1:**
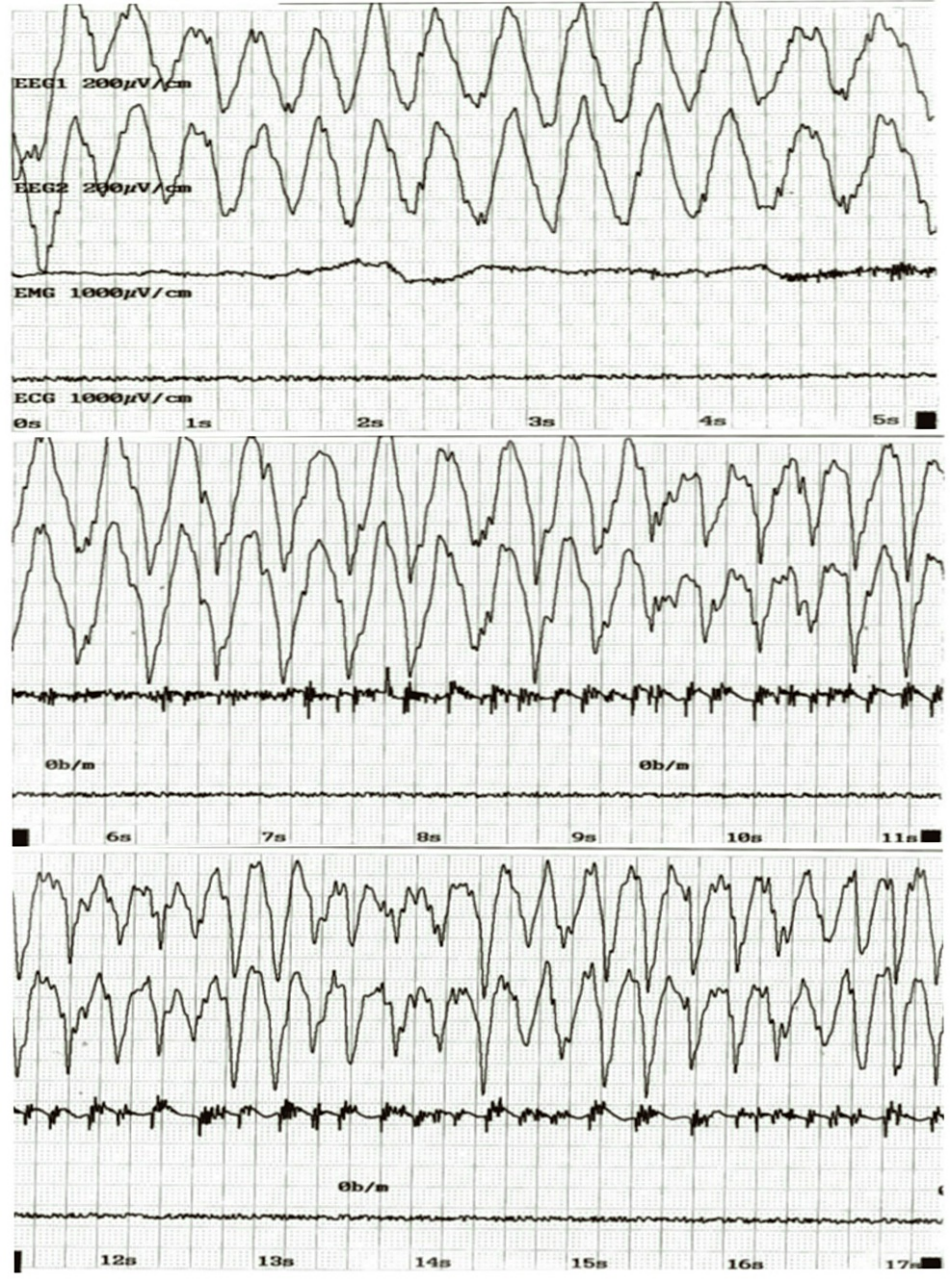
Electroencephalography of the patient. A routine 20-minute video electroencephalography (EEG) shows no focal slowing, no interictal epileptiform discharges, and no electrographic seizures.

Lumbar puncture, cerebrospinal fluid (CSF) studies, and electroencephalogram (EEG) were also within normal limits, as shown in Table 2 and Figure 1.

**Table 2:** Cerebrospinal fluid (CSF) study results. CSF: Cerebrospinal fluid; mg/dL: milligrams per deciliter; RT-PCR: Reverse transcription polymerase chain reaction.

CSF Study	Reference Range and Units	Results
Spinal Fluid RBC	0 Cells/mm3	0 Cells/mm3
WBC, CSF	0 Cells/mm3	0 Cells/mm3
Protein, CSF	15-45 mg/dL	33 mg/dL
Glucose, CSF	45-70 mg/dL	66 mg/dL
Culture, CSF	Negative	No aerobic or anaerobic growth seen
Cryptococcal Antigen	Negative	Negative
Enterovirus by RT-PCR	Negative	Negative

The urine drug screen was abnormal and was positive for cannabis (Table 3).

**Table 3:** Urine drug screen of the patient. LC-MS-MS: Liquid chromatography with tandem mass spectrometry.

Component	Reference Range and Units	Results
Amphetamines	NEGATIVE	NEGATIVE
Benzodiazepines	NEGATIVE	NEGATIVE
Cannabinoids	NEGATIVE	POSITIVE
Cocaine metabolite	NEGATIVE	NEGATIVE
Hydrocodone	NEGATIVE	NEGATIVE
Morphine/Codeine	NEGATIVE	NEGATIVE
Oxycodone	NEGATIVE	NEGATIVE
Methadone metabolite	NEGATIVE	NEGATIVE
Quantitative analysis by LC-MS-MS		
Cannabinoids	NEGATIVE ng/mL	>400.0 ng/mL

There was a history of extensive cannabis use, with the patient consuming 4 grams daily, made available to him through his "medical marijuana card." He experienced his first psychotic episode, which included paranoid delusions and hallucinations, at age 16 after smoking cannabis, and it progressed to severe catatonia. According to his mother, he stopped using marijuana for a few months following that episode and did not exhibit any psychiatric symptoms during that time. He never used synthetic drugs or other illicit drugs. He restarted his use of marijuana six months ago after a fight with his girlfriend. He was using marijuana two-to-three times a week. His mother reported that two weeks ago, he had a breakup with his girlfriend and started using four grams of marijuana every day, and he started isolating himself in the room. During this episode, he did not experience any delusions, auditory or visual hallucinations. His mother became concerned when he isolated himself, stopped eating and drinking, and subsequently brought him to the ER. There is no family history of psychiatric illness or alcohol or drug use. Given the acute onset of catatonic symptoms following cannabis use, a diagnosis of cannabis-induced catatonia was suspected, and the patient was admitted to the psychiatric unit for further evaluation. The patient began exhibiting mixed episodes of retarded and excited catatonia. Treatment with lorazepam 2 mg IV and liquid diazepam 10 mg orally four times daily was administered for three days, but his symptoms and Bush-Francis Catatonia Rating Scale scores did not improve. Considering the treatment-resistant nature of the patient's condition, electroconvulsive therapy (ECT) was discussed as a therapeutic option. With informed consent from the patient's legal guardians, a course of bilateral ECT was commenced. The patient underwent 10 ECT sessions over a span of four weeks. Concurrently, he was started on quetiapine 25 mg twice daily and 100 mg at night, along with lorazepam 2 mg three times daily for maintenance.

Results

Following the third ECT session, the patient exhibited a notable improvement in his catatonic symptoms. The vacant gaze and mutism gradually resolved, making him more responsive. Over time, the patient became increasingly responsive to external stimuli as these symptoms subsided. By the tenth ECT session, the patient's rigidity had significantly decreased, and both his speech and voluntary movement had almost fully recovered, as detailed in Table 4. After being discharged from the hospital, the patient was enrolled in an outpatient program for marijuana addiction treatment, and he also began individual therapy. He was scheduled for ongoing outpatient psychiatric follow-up.

**Table 4:** Treatment progress and symptom resolution in a patient with catatonia. ECT: Electroconvulsive therapy; TID: Three times a day; PO: Per oral (by mouth); BID: Twice a day; HS: At bedtime (Hora Somni); mg: Milligram.

Weeks	ECT sessions/week	Medications	Symptoms
Week 1	3	Lorazepam 2 mg TID PO quetiapine 25 mg TID PO	Vacant gaze, catalepsy, limb rigidity, waxy flexibility, mutism and negativism
Week 2	3	Lorazepam 2 mg TID PO quetiapine 25 mg BID and 50 mg HS PO	Vacant gaze and mutism resolved. More responsive to external stimuli
Week 3	2	Lorazepam 2mg TID PO quetiapine 25 mg BID and 100 mg HS PO	Limb rigidity and negativism improved
Week 4	2	Lorazepam 2 mg TID PO quetiapine 25 mg Bid and 100 mg HS PO	Speech and voluntary movement fully recovered. Catatonia resolved

## Discussion

This case highlights the potential association between cannabis use and the development of catatonic symptoms in susceptible individuals. While the precise pathophysiology of cannabis-induced catatonia remains unclear, it is hypothesized that the psychoactive compounds in cannabis may interact with neurotransmitter systems implicated in catatonia. As the legalization and use of cannabis products are growing, understanding these interactions becomes critical. Cannabis comes in a variety of forms and has varying potency, determined by the concentration (%) of 9-tetrahydrocannabinol (THC), the major psychoactive component of the cannabis plant. Although catatonia lacks specific pathophysiological mechanisms, evidence in the literature suggests that it occurs primarily due to decreased activity of the GABA-A receptor, dopamine at the D2 receptor, and glutamate at the N-methyl-D-aspartate (NMDA) receptor [5,6]. Benzodiazepines (BZDs), particularly lorazepam, are the first line of treatment for catatonia, as they enhance GABA receptor activation while lowering NMDA receptor activity [7,8]. ECT is effective for patients unresponsive to BZDs [4,8]. Avoiding aggravating factors such as recreational or illegal drug use and dopamine-blocking medications may help reduce catatonia symptoms and prevent recurrence [4]. Distinguishing between cannabis-related psychosis and schizophrenia can be challenging; however, they can often be differentiated by shorter episode durations, onset after substantial cannabis use, and the absence of psychotic symptoms if the substance use has not relapsed.

Although there is limited literature and evidence available for cannabis-induced catatonia, the increasing prevalence of cannabis use disorder (from 2.9% to 30.6%) shows that physicians should consider cannabis as the probable etiology of catatonia in the future [6]. Furthermore, catatonia is an unpleasant and traumatic experience for the patient; physicians should be cautious to recognize and treat it as soon as possible [9]. Extra consideration should be given to the likely adverse effects of marijuana. Managing cannabis-induced catatonia is challenging, and treatment options are limited [5]. Pharmacological interventions, including benzodiazepines, antipsychotics, and mood stabilizers, are commonly employed but may not always yield satisfactory results. In such cases, as demonstrated in this report, ECT can be a valuable and effective treatment modality for refractory catatonia [8].

## Conclusions

In our patient, the factors supporting a diagnosis of cannabis-related catatonia include the first onset of a psychotic episode coinciding with the commencement of cannabis smoking, no previous psychiatric history, no family history of psychiatric disorder, and heavy use of cannabis leading to catatonia. Normal blood and CSF test results, an MRI of the brain, an EEG, and a neurology consultation all ruled out neurodegenerative psychosis. Therefore, in the absence of any other etiology, it was concluded that the patient's catatonic presentation was attributable to his cannabis use. Physicians should exercise vigilance when prescribing medical and legalized cannabis. Clinicians should also maintain a high index of suspicion for catatonia in patients presenting with acute-onset catatonic symptoms following cannabis consumption. This case report emphasizes the importance of considering ECT as a treatment option for individuals resistant to conventional pharmacotherapy.
